# Neuroinflammation and Neurodegeneration of the Central Nervous System from Air Pollutants: A Scoping Review

**DOI:** 10.3390/toxics10110666

**Published:** 2022-11-06

**Authors:** Frances Vivienne Armas, Amedeo D’Angiulli

**Affiliations:** 1 Department of Biology, Carleton University, Ottawa, ON K1S 5B6, Canada; 2 Department of Neuroscience, Carleton University, Ottawa, ON K1S 5B6, Canada

**Keywords:** neurotoxicity, developmental neurotoxicants, particulate matter, neurodegenerative diseases, neuronal apoptosis, neuroinflammation, hormesis, vitagene network

## Abstract

In this scoping review, we provide a selective mapping of the global literature on the effects of air pollution on the life-span development of the central nervous system. Our synthesis first defines developmental neurotoxicants and the model effects of particulate matter. We then discuss air pollution as a test bench for neurotoxicants, including animal models, the framework of systemic inflammation in all affected organs of the body, and the cascade effects on the developing brain, with the most prevalent neurological structural and functional outcomes. Specifically, we focus on evidence on magnetic resonance imaging and neurodegenerative diseases, and the links between neuronal apoptosis and inflammation. There is evidence of a developmental continuity of outcomes and effects that can be observed from utero to aging due to severe or significant exposure to neurotoxicants. These substances alter the normal trajectory of neurological aging in a propulsive way towards a significantly higher rate of acceleration than what is expected if our atmosphere were less polluted. The major aggravating role of this neurodegenerative process is linked with the complex action of neuroinflammation. However, most recent evidence learned from research on the effects of COVID-19 lockdowns around the world suggests that a short-term drastic improvement in the air we breathe is still possible. Moreover, the study of mitohormesis and vitagenes is an emerging area of research interest in anti-inflammatory and antidegenerative therapeutics, which may have enormous promise in combatting the deleterious effects of air pollution through pharmacological and dietary interventions.

## 1. Introduction

In recent years, the development of urbanized and industrialized communities has been in constant and incessant expansion. Predominant examples of this process can be found in megacities such as the metropolitan area of Mexico City (MCMA) and in major U.S. cities such as New York City (NYC), which constitute the geographical reference framework for our work. Air pollution in these and many other areas around the globe is enormously increasing due to various factors that are not yet properly regulated and rationalized, such as, more specifically, vehicle emissions, industrial plants, the use of commercial chemicals, and domestic emissions such as cooking and heating, etc. Furthermore, highly industrialized metropolises inevitably entail an increase in population density per area or spatial settlement area. For example, MCMA has a population of around 24 million while [1] NYC has a current population of around 8 million [2]. However, it is also necessary to mention other cases such as Delhi, India, and especially Jakarta, Indonesia, which in the last decade has been in the lead in monotonic increase in air pollution per capita [3], as well as cases of non-metropolitan districts or more rural regions such as various areas of Mongolia [4], where coal-based combustion for heating, cooking, and food preparation eliminates the traditional distinction between indoor and outdoor pollution. The growing number of habitants in these key urban areas is of concern in life cycle health studies, particularly in children and infants, as it has been rather conclusively shown that substances in highly polluted and industrialized regions have a high probability to affect the neurological development of a significant fraction of the pediatric populations and can cause neurobehavioral deficits. For instance, and as a quick preview, outdoor airborne particles have been associated in children with cases of attention deficit hyperactivity disorder (ADHD), autism spectrum disorder (ASD), and other developmental neurological disorders [5]. At the opposite end of the continuum of the developmental cycle, that is, in the elderly, there is undeniable probabilistic continuity of negative neurological outcomes connected with many or all the manifestations of neurodegenerative conditions typically associated with various dementias, including Alzheimer’s disease (AD) and other devastating conditions such as Parkinson’s disease (PD). This developmental continuity describes the approach proposed in this review, where the decline in neurocognitive functions at the end of adult life is intimately connected with neurological ontological development at the beginning of this life cycle, and where the main thesis is the working hypothesis that the toxic agents polluting the airways have the main effect of altering the normal trajectory of neurological aging in a propulsive way towards a significantly higher rate of acceleration than that which could be expected if the atmosphere were much less polluted. It is also hypothesized that the major aggravating role of this neurodegenerative process is mainly due to the complex action of neuroinflammation, which not only prevents but also actively counteracts protective factors of self-repair and self-regeneration which, within certain margins, could compensate and delay the aging of the neural tissues and their possible necrosis.

In the synthesis presented here, we seek to clarify the direct role airborne pollutants play in children’s brain health through evidence that aims to delineate neurodevelopmental outcomes, behavioural deficits, and neurological impairments. Following current medical research standards [6], our broad *scoping review* renders various evidence from a large body of multidisciplinary literature that focuses on developmental neurotoxicity, as well as an overview of key ideas, arguments, hypothesized mechanisms, and principles that contribute a unitary understanding of the influence of air pollutants on the developing CNS and its functions. More specifically, particulate concentrations in megacities and the several possible mechanisms underlying neuroinflammation following significant and severe exposures to these substances are discussed in detail. Finally, we conclude by linking the developmental processes in children with possible associated neuropathological consequences in adults and the elderly. Additionally, we sketch some points of entry for action, based on very recent scientific data, to remedy the current situation in terms of prevention and precautionary interventions in order to protect and safeguard healthy neurological development in future generations, locally and globally.

## 2. Developmental Neurotoxicants and Model Effects of Particulate Matter, Fine Particulate Matter, and Ultrafine Particulate Matter

Neurotoxicants can be defined as types of chemicals known to induce adverse effects on the nervous system following exposure during CNS development throughout the life cycle, often known as developmental neurotoxicity (DNT) [7]. These substances can range from industrial chemicals to organic solvents and pharmaceuticals. In addition to their neurotoxic repercussions, neurotoxicants are inherently complex to define, as they involve a dynamic and complex convolution (correlated with the time–historical dimension) when it comes to approximating the amount of exposure or trying to break them down more analytically, that is, to establish their “dosage” in an ecological context by decomposing complex and global exposure into units of exposure to a single substance. One of the consequences of this complex dynamic is that the number of identified neurotoxicants cannot at all be established in a final and conclusive way; on the contrary, it grows continuously every year. For example, a dozen new substances toxic to the human nervous system were discovered between 2006 and 2013, for a total of approximately 214 neurotoxicants [8]. Since the time of that estimate, it is highly probable that this convolutive summation has changed as new products are constantly being introduced and sold in commercial businesses. A recent estimate has proposed that around 30,000 chemicals, or around 30% of all substances used commercially, have the potential to be neurotoxic [9].

Scientists are constantly monitoring this number, mainly because presumably if we take into account experimental laboratory studies, about 1000 chemicals are neurotoxic to animals, suggesting then that there may be others that are lethal to humans. Maffini and Neltner identified over 300 chemicals contained and allowed in foods that have detrimental effects on human brain development [10]. Pesticides are the subgroup that accounts for most of the official neurotoxicants’ total number [8].

The experimentation investigating new dangerous substances for the CNS has been accelerated in recent years owing to the clear elucidation of their harmful properties. However, significant efforts are still underway to create faster assessments and determinations of potential adult neurotoxicants. Specific guidelines are established by the United States Environmental Protection Agency (USEPA) and the Organization for Economic Co-operation and Development (OECD) to improve our understanding of the potential neurotoxic characteristics of certain substances [11]. The tools at our disposal include clinical assessments and observations ranging from measuring motor activity to clinical and neuropathological assessments. These tests underlying the OECD guidelines are meant to dispense a primary tier of screening which would be followed by further assessments once positive findings were to be found [10]. Multilevel testing from guidelines of various organizations poses a certain degree of criticality and importance in experimentation as they provide a deeper insight into a chemical’s potential neurotoxicity that is further than what is detected in standard testing of toxicity. Numerous studies have shown that neurotoxicants interfere during various stages of biological processes such as cell migration and proliferation. Additionally, the developing nervous system of infants and children is more vulnerable to these substances. Molecular alterations in the nervous system strongly affect the structure and function of the brain. This creates an association between neurotoxicant exposure to various neurodevelopmental disabilities such as mental and intellectual developmental disorders, attention deficit hyperactivity disorder (ADHD), autism spectrum disorder (ASD), etc. [11]. Data from a U.S. survey by Grandjean and colleagues revealed that industrial chemicals which induce neurotoxicity are associated with a dramatic loss of intelligence quotient (IQ) due to neurodevelopmental disorders [9]. That said, the academic achievement and socioeconomic outcomes of children and their families are also strongly influenced, which is why the number of studies investigating associations between neurotoxicants and developmental outcomes has increased exponentially in recent years.

**Figure 1:**
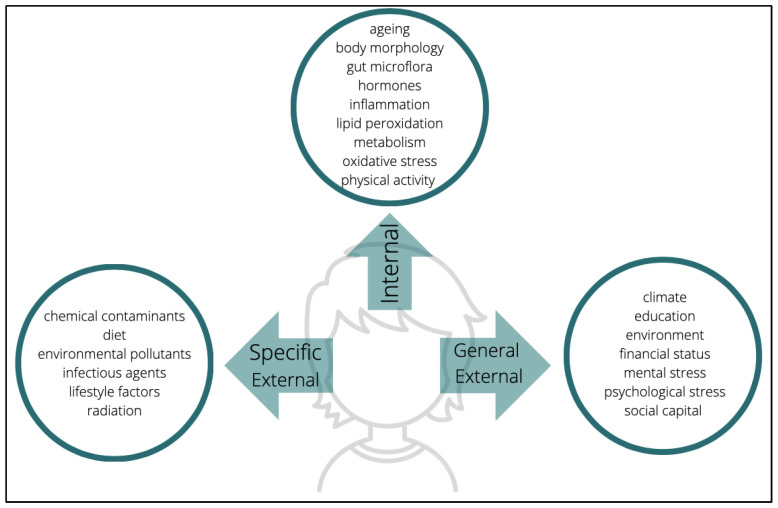
Three domains of exposome with narrow examples underlying each domain. Graphical diagram was adopted from [18] with permission from Oxford University Press, 2022.

In addition to the neurotoxicants that are mainly present in, or made up of, the materials that surround us daily, there is also the problem of small particles that are found in the air due to emission or combustion pollutants. Particulate matter (PM) is a complex compound of solid and liquid particles emitted by vehicles, industrial plants, power plants, residential heating systems, etc. [12]. PMs are further divided into categories based on the diameter of a particle. For example, PM_10_ are particles smaller than 10 micro millimeters (µM) in diameter that are also typically contained in traffic-related air pollution [12]. Furthermore, air molecules that have an aerodynamic diameter <100 nano millimeters (nm) are considered ultrafine particulate matter (UFPM). Both PM and UFPM are present within indoor and outdoor environments, which raises a critical issue, as either can act as neurotoxicants and are equally linked to neurological disorders [7,13]. According to an estimate by the World Health Organization (WHO), around 3.1 million deaths in 2010 were caused by ambient air pollution [14]. To add to this data, exposure to fine particles, PM_2.5_, was found to reduce life expectancy by an approximate average of 8.6 months [14]. These incidences are strongly associated with the size and intensity of characteristics of PM and UFPM that allow them to remain in the air and cross the blood–brain barrier, gastrointestinal tract, respiratory structures, and other major body systems (mechanisms which we describe in more detail afterwards). Numerous investigations examine the probable role of neurotoxicants in inducing neuroinflammation, in particular the mechanisms and biomolecular alterations that occur after exposure. The most common assessment includes experimental exposure of cells to neurotoxicants such as PM, which is one of the most used and well-known models in this area of study. The laboratory results suggest a common distinguishing feature among many diseases associated with air pollutants: inflammation [15]. The research literature summaries propose various possible pathways taken by neurotoxicant particles within the body. For example, PM has been shown to cause inflammatory responses by invading mainly the epithelial cells of the respiratory structures of the airways [15]. As part of the body’s natural immune response, oxidative stress and the production of reactive oxygen species (ROS) are some of the consequences of proinflammatory mediators. Following chronic exposure, molecules such as ROS subsequently cause tissue damage while oxidative stress indicates neurodegeneration as shown by animal models [15,16] (see below). These are just some of the more common molecular alterations in the body after being subjected to neurotoxicants. In consideration of the size and the overwhelming ability of these particles to instigate various changes, the number of probable paths that could be followed within the body, in organs, and especially in the CNS, by neurotoxicants is still undefined. Not only is their influence based on intrinsic characteristics, but many other possible interacting factors also contribute to their mechanism of action. Specifically, the duration and intensity of exposure to neurotoxicants determine the degree of alteration in the body [17]. According to the conceptualization offered by the so-called construct of exposome, the measurement of exposure depends on three domains: general external environments, specific external, and internal (Figure 1) [18]. The general external embodies psychological and socioeconomic influences such as stress, level of education, climate, etc. On the other hand, the specific external is restricted to lifestyle choices and exposure to pollutants; alcohol consumption, tobacco consumption, diet, exposure to radiation, contaminants, and pathogens, etc. are the basis of this domain. Finally, the internal environment is composed of endogenous factors such as an individual’s metabolism, oxidative stress, intestinal microflora, hormonal status, and other more organic and genetic individual characteristics. Undeniably, these examples are non-exhaustive; they are only among some of the recurrent exposures to which individuals may be subjected to. From conception to death, there are multifarious factors that will cumulatively characterize a person’s exposome subjection. The myriad elements have become a challenge when it comes to measuring the exposome as it seems unthinkable to assess each one that underlies every domain. However, targeting this issue can start off with an evaluation of available tools which can be used to measure exposomes. For an instance, modern technologies such as mobile phones can be used as sensor devices to monitor physical activity, stress, heart rate, sleep schedule, etc. Another approach is the utilization of biomarkers and tools such as epigenomics, transcriptomics, and other omics which exclusively target the assessment of components underlying the internal domain [18]. Wild [18] also suggested that there may be circumstances in which several factors overlap with each other, either within the same domain, or across different domains, or in all given possible combinations; however, one thing is certain: the degree of an exposome is reflected in these three domains. This is to say that, with the additional support of many other types of evidence and inferences, it is safe to speculate that some individuals belonging to cohorts that reside in megacities are more vulnerable to neurotoxicants than cohorts residing in other types of urban or rural ecologies.

Another problem that is associated with the study of neurotoxicants is the limited knowledge about most chemicals. The vast majority of the literature dealing with neurotoxicants focuses on PM and other components of common organic compounds. The amount of information provided on these articles and investigations is in every way relevant and constructive, however, the extent to which numerous other chemicals should be equally studied is substantial. Therefore, we cannot really establish conclusions about neurotoxicants that are generalizable to all environmental situations or that offer a comprehensive taxonomy of mechanisms. For example, extensive literature examines the consequences of PM in the body. PM is a common DNT test bed because it is consistently and continuously present in the air of polluted areas, which creates an easier and more consistent system in terms of the effect of measurements on individuals living in the community who had already been exposed to it in the long term. However, the exposure is moderate or more scattered for other neurotoxicants such as heavy metals.

Heavy metals, along with other contaminants, while being omnipresent in the products we use, in the food and water we consume, and in our environment, present the problem of a more complex dosage in terms of exposure. Some of the most common types of heavy metals are arsenic (As), cadmium (Cd), lead (Pb), and mercury (Hg). There is a vast literature that discusses the toxicity of heavy metals which often range from digestive and respiratory repercussions to neurodevelopmental implications. As an example, the evidence presented in Table 1 contains a few of the recently published articles that provide some insights on the toxicity of four heavy metal types. Toxicological assessments provide some evidence that suggests their role in the intensification of oxidative stress and the production of ROS in the body; concurrently, neuronal function and the release of neurotransmitters also show perturbations that can systematically lead to neurodevelopmental disorders [19].

Even considering the incompleteness and limitations of our knowledge base, incompleteness does not necessarily imply that the links of associations and causal actions established so far are inconclusive. Instead, what is legitimate to conclude at present is that, even if we do not exactly know the most minute details in terms of single substances, there is a certain connection between environmental pollution, especially air pollution, and irreversible perturbative or dissipative processes of neurobiological structures and functions. These perturbations run in the same parallel temporal direction as the ontogenetic developmental plasticity processes.

It seems quite plausible that while the diversity of adverse conditions associated with neurotoxicants is complex and ever changing, restricting the study to people living in megacities is a major step toward advancing our scientific understanding of environmental neurodevelopmental disorders. However, a persistent gap needs to be bridged when it comes to different demographic groups. Despite assumed parity of conditions, other variables also create an apparent difference between the subgroups. A construct mentioned in several articles alludes to socioeconomic status (SES) as a latent variable (or more precisely covariate) that is linked to chemical exposure. As already mentioned, the SES is generally a composite construct that incorporates factors such as occupation, race/ethnicity, income, parental education, residence, and other variables [27]. SES is thought to be a background correlate of children’s exposure to certain chemicals [28]. Susceptibility to neurotoxicants may be due to several factors, and the hypothesis that poverty and/or low individual or familial SES may in themselves be a neurotoxic vulnerability index is still vigorously debated. Much less controversial, however, is the observation that SES can serve as a more global index of socioenvironmental risk, which in fact incorporates air pollution. In the next section, air pollution is discussed as a model test bed for identifying the neurotoxic outcomes of these lethal particles.

## 3. Air Pollution as a Test Bed for DNTs

Air pollution is one of the central and most common elements used to test the neurotoxicity of numerous neurotoxicants. Pollutants in the air are made up of mixtures of solid and liquid particles ranging from metals to gases. Ground-level ozone (O_3_), sulphur dioxide (SO_2_), carbon monoxide (CO), nitrogen oxides (NO), polycyclic aromatic hydrocarbons (PAH), lead, and other heavy metals are all suspended in the air, which raise concern for public health [29]. These pollutants are also considered as particulate matter that can potentially induce toxicity. The increase in pollutant concentration is related to the extent of urbanization and industrialization. Indeed, the main proven sources of air pollution are industry, agriculture, dust, biomass, and the combustion of fossil fuels [29]. The aerodynamic diameter of the particulate matter establishes relevance in our review of the findings as it becomes apparent that the power of neurotoxicants is inversely proportional to their size.

In 2015, data showed that 8.9 million deaths were caused by ambient air pollution from PM_2.5_ [30]. PM_2.5_ is now considered to have a major impact on the death rate worldwide. The World Health Organization recently estimated that around 91% of the population in low- and middle-income countries (or in other words, low-SES countries) is prone to outdoor air pollution [31]. Among these data, there are also 4.2 million premature deaths. Cancer is one of the predominant contributors to this number, which is why PM is now labelled as a carcinogen. According to WHO, fine particulate matter (PM_2.5_) has a guide value for air quality of 5 μg/m^3^ on an annual average and 15 μg/m^3^ on average over 24 h, while coarse particulate matter (PM_10_) has an annual 15 μg/m^3^ and an average of 45 μg/m^3^ over 24 h [31]. Calderón-Garcidueñas and colleagues previously stated that in three cities in Mexico—Xalostoc, Merced, and Pedregal—the average of PM_10_ over 24 h was recorded at 75 μg/m^3^ [1]. These data exceed both WHO and USEPA guideline values. On the other hand, a survey of European countries shows Albania as the nation with the annual average per population of the highest level of PM_10_ of about 81 μg/m^3^ [14]. As in these examples, geographic distribution surveys on pollutant concentrations strongly exemplify how low- and middle-SES countries have higher percentages of cities that consistently fail to meet WHO air quality guidelines, compared with countries with high SES.

Despite the environmental awareness fostered by scientists, activists, and non-governmental organizations, it is no secret that urban pollution is an increasing global problem. As a result, the incidence of pollution-associated diseases is also expected to dramatically increase, as is the death rate associated with these diseases. The following section focuses on the effects of neurotoxicants in animal models which contribute to elucidate, in a more experimentally controlled way, the possible neuropathophysiological mechanisms involved in exposure to environmental pollution.

## 4. Animal Models and DNT

Similar to other laboratory investigations, animal models are used in studies related to the neurotoxicity of chemical compounds. Experiments which focus on neurotoxicant exposure in rodents have become a backbone that allows researchers to draw conclusions on the human viewpoint of the situation, given that various hierarchical cascades and mechanisms of diseases in rodents ensue quite similarly (i.e., they are homologous) in humans, due to homologous physiological and anatomical facets. Conveniently, rodents can also be genetically manipulated and are widely available through laboratory distributors.

However, the question of ethicality is often and recurrently raised in societal and political settings where some researchers argue against animal experimentation. For one, in a recently published article, Palloca and Leist [32] have argued that certain parameters are set to determine the usefulness of animal models in many countries but while doing so, researchers reason with strong reification or the assumption that animal models are widely used because of the similarity in behaviour and biological reactions that they share with humans, mistaking ideal models with reality. Although these arguments could be valid for a portion of animal models, they would not necessarily apply to *all* animal models or research. Many excellent instances of valid animal model toxicology test studies do exist, especially those which seek to elucidate probabilistic links between the findings and outcomes in animals and humans, and those which seek to translate some aspects across the two contexts. Another important point made by Palloca and Leist is that in toxicology, toxicity often has numerous causes and consequences which require delineation not by one, but by many methods [32]. As such, alternatives to animal models can be used to target each method. For example, the availability of stem and progenitor cells has now constructed a novel alternative way of testing for DNT [33]. Although this method has been successful in demonstrating the consequences of neurotoxicants in many key events such as apoptosis, neural proliferation, neuronal migration, and others, it is still regarded to be at an early state. As a result, other crucial events such as neuronal maturation, glia differentiation, and others are not covered. In addition, the most critical (and paradoxical) issue with the use of stem and progenitor cell DNT testing is the lack of coverage that it offers when it comes to observing the effects of neurotoxicants in the emotional and intellectual consciousness of humans [30]. Thus, in spite of the fact that the bandwidth of advantages this method already shows to offer is quite promising, it is still in progress. All things considered, animal models are, at least for now, an indispensable tool to tackle DNT. Accordingly, we review a few selected examples of studies which provided insights that are translatable to the human developmental context.

A laboratory investigation performed on mouse models shows that exposure to environmental ultrafine particles during the third trimester of pregnancy and 4–7 days after birth resulted in DNT that is similar to the observable characteristics of ASDs [34]. In addition to PM, diesel engine exhaust (DEE) also greatly affects the central nervous system of transgenic mice. DEE exposure appears to impair motor function and accelerate plaque formation in mouse models [35]. This then results to be associated with the development of AD, particularly from long-term exposure. In another study, PM_2.5_ was shown to reduce the memory and spatial learning difficulties of rodents [36]. This current research proposes that the absorption of PM_2.5_ after inhalation induces morphological and physiological alterations. Promoting changes in molecular processes can hinder neurodevelopmental mechanisms such as synaptogenesis [36]. Due to these intrusions, rodents tend to exhibit depressive behaviors. To add to the growing evidence of disorders, an investigation that subjected young male F1 mice to PM_2.5_ led to anxiety-related behaviors that appear to be associated with reduced plasma levels of interleukin-18 and vascular endothelial growth factor [37]. The wide variety of articles discussing DNT on the developing CNS of rodents suggests how complex these particles are. The evidence from animal studies of DNT presents environments with varied exposure conditions to neurotoxicants. In the three referenced articles, it is clear that subjecting animal models to different kinds of toxicants ensues a certain degree of DNT. This pattern of consequences parallelizes with evidence observed in adults and children who develop DNT after varying levels of exposure, as seen in subsequent sections of this review.

Excluding neurodevelopmental diseases attributable to other factors, neurotoxicants alone have been demonstrated to be associated with significant changes in the neurological health conditions of animal models such as the examples of studies selected for this discussion. The disparity in behaviours between exposed and unexposed individuals contributes enormously to our understanding of DNT. Even more important is the idea that exposure to neurotoxicants in the prenatal phase already creates dramatic influences on the fetus and the newborn, which more critically places the neurotoxicant class at the top as one of the highest health risks. The available evidence also strongly suggests that neurotoxicants should be more widely studied using a developmental and longitudinal perspective.

## 5. DNT-Related Systemic Inflammation in Children

The previously ascertained deleterious effects of neurotoxicants using animal models are fundamental in view of the fact that behavioural outcomes are very similar to the observed consequences in children. These similarities allow the plausibility in stating that the effects of neurotoxicants on the developing brain of animals mirror possible mechanistic models of the action of these particles on the human brain. This section examines the most recent evidence regarding neurotoxicity in children residing in megacities such as the MCMA. Some plausible hypotheses on the mechanisms of action and the hierarchical cascade triggered by neurotoxicants within the developing brain are also reviewed.

Most of the literature summaries state that air pollution indicators primarily affect the central nervous system (CNS). Mechanically, it is mainly inhaled or ingested, and the aerodynamic diameter of the particle determines its path within the body. Coarse particulate matter tends to remain suspended in the upper airways while PM_2.5_ ends up in the lungs with a certain proportion distributed in the throat [38]. Due to its size, PM_2.5_ can also invade the olfactory epithelium and reach the brain via the olfactory cortex [39]. In addition to the brain, the PM can also reach other organs through translocation from the lungs to the blood [40]. Blood-contained neurotoxicants are spread throughout the body from which they can be distributed anywhere, including regions of the brain. This path is one of the prime examples of why large rates of deaths in 2015 were due to air pollutants causing cardiovascular disease, stroke, and lung cancer [40]. Unfortunately, children are more vulnerable to neurotoxicity than adults.

As is known, babies absorb more water and air per unit of body size than adults [7]. In addition, they spend more time playing outdoors and being exposed to dust particles. These variables contribute to the increased likelihood of risk and promote vulnerability to neurotoxicants. A modest number of studies mention these environmental variables as risk factors specific to children, while the greatest attention is centered on their intrinsic immaturity of the immune and nervous systems. Neurological and plasticity development are important phases that occur especially during early childhood. This is the period in which myelination, cell proliferation and migration, the construction of neuronal connections, etc. are taking place [41]. That said, brain development depends on the progression and success of these processes, which is why clean air is a necessity. Even a slight change in environmental conditions can have deleterious impacts on these complex events that could potentially evolve into a permanent condition.

**Figure 2:**
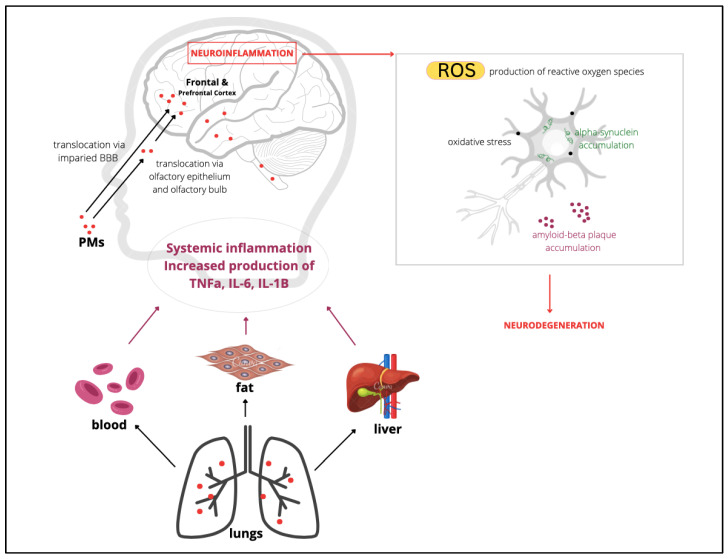
Example of mechanisms of action leading to developmental neurotoxicity from inhalation of particulate matter. Note: TNFa, tumor necrosis factor alpha; IL-6, interleukin-6; IL-1B, interleukin-1 beta; BBB, blood–brain barrier; ROS, reactive oxygen species. Reused with author’s permission, from [5].

A substantial body of scientific research converges on a similar central notion that systemic inflammation begins with the disintegration of the barriers that support our functional organs. More critically, this system also induces neuroinflammation following activation of microglia associated with impaired blood–brain barrier (BBB) [12]. Inhalation leads to the absorption of PM and metal particles by olfactory neurons and cranial nerves [1]. It is very important to note, however, that the entry point of neurotoxicants does not occur exclusively through the nasal or oral air passages by respiratory inhalation. Neurotoxicants can also impair the body through ingestion. Ingested particles tend to follow an independent path in which they disrupt the mucosal barrier of the gastrointestinal (GI) tract and so-called tight junctions (TJs) in various body organ systems [1]. Many experimental studies have shown that abdominal pain and inflammatory bowel disease are associated with the breakdown of the gastrointestinal tract barrier due to air pollution [42,43]. Specifically, in experimental studies on dogs from Mexico City, it was found that their gastrointestinal tract is in fact weakened by exposure to PM [12]. This creates a critical assumption that the gastrointestinal tract of children in the MCMA is also most likely compromised. Over the years, increasing evidence of bowel disease has been reported in highly polluted countries. A recent systematic summary by Vignal and colleagues [44], which focuses on intestinal diseases due to pollutants, reported that exposure to pollutants such as PM_2.5_, CO, and NO during the prenatal and infant stages is positively correlated with inflammatory bowel disease (IBD). Various forms of cancer, such as colorectal, anal, and small bowel cancer, have also been associated with exposure to exhaust emissions. The range of the duration of exposure of individuals who have experienced IBD is rather wide. However, a surprising finding is that the incidence derived from hospitalization accounted for diseases of the digestive system in a single day of exposure in patients who were monitored during recurring hospital admissions initially due to other causes. This observation suggests that PM is powerful enough to instigate damage to the digestive system even after such a short duration of exposure [45]. Additionally, the enteric nervous system is thought to participate during the periods of CNS vulnerability caused by neurotoxicants, as higher antibodies have been discovered in children living in the MCMA, which function directly for neural proteins and cell junction [6]. In addition to the consequences of ingested PM, heavy metals also appear to induce damage to the digestive system. Over the years, there has been a steady increase in heavy metal pollution in various countries around the world. The most common heavy metals are arsenic contained in drinking water while cadmium, lead, and mercury are components of many baby foods [46]. These toxic substances have recently been extracted in parallel with alterations in the metabolic profile of the intestinal microbiota. In a normal functional system, our gut microbiota is critically important for regulating digestion and playing a role in our immune system. In addition, it is also responsible for the metabolism of a variety of substances, as seen in rodent experiments, in which heavy metal accumulation and absorption was decreased in individuals who had a functional gut microbiota and had not been treated with antibiotics nor were free of germs due to antibiotic treatment. The metabolization of these substances follows the process of excretion which further reduces an individual’s exposure to toxicity. Regardless, heavy metals were observed to target the gut microbiota and induce changes in its composition in a review published by Duan and colleagues [46]. For one, a wide range of evidence presents observations pointing towards heavy metal binding on the reduced growth of bacteria in the gut through disruptions in protein synthesis. Without microorganisms, the ability of the intestine to metabolize ingested particles is impaired, leading to a progressive development of metabolic diseases [46]. Currently, very limited information is available on the effects of heavy metals on the digestive system and gut microbiota as most studies pay more attention to their influences in other systems such as neurological functions (Figure 2).

Impairment of lung function is as serious as digestive problems caused by neurotoxicants. In Salamanca, Mexico, Linares and colleagues determined that schools built closer to major stationary sources of air pollution have higher percentages of students with abnormal respiratory system function than those who come from distant schools [47]. Most studies have now confirmed that PM and UFPM cause inflammation of the respiratory tract of children. Adversely, activation of the innate immune system as part of the body’s immune response also causes the circulation of inflammatory mediators such as the production of endothelin-1 (ET-1), a peptide that regulates vascular smooth muscle tone and acts as a vasoconstrictor [48]. This systemic inflammation results from an increase in cases of poor vagal response and altered heart rhythm in children within the MCMA who are constantly exposed to air pollutants. One considered logical pathway that allows PM to reach the brain includes the respiratory system through inflammation mediators. As a further example, the production of interleukin-1β, tumor necrosis factor (TNF), and interleukin-6 (IL6) compromises brain regions due to the fact that receptors for these mediators are expressed in the brain blood vessels [49]. Cases of lung infections and diseases have not only proved to be concentrated in the MCMA, but the sources of evidence have also extended to megacities in other countries known to be highly urbanized. A recent study conducted in Poland evaluated 1475 children who were isolated in three distinct locations with varying concentrations of PM_2.5_ or PM_10_ [50]. The study was based on a questionnaire from which the sociodemographic characteristics and symptoms of each child were compared. The main finding is that the average exposure to PM in children resulted in an increase in reports of upper respiratory tract symptoms such as runny nose, respiratory fatigue, coughing, sneezing, and nasal congestion [50]. Beyond similar symptoms, a survey in Shenyang, China, has provided a strong link between outdoor pollution and increased mortality from acute lower respiratory tract infections [51]. In the latter study, the pollutants identified were PM_2.5_, PM_10_, SO_2_, NO_2_, and O_3_. Guo and colleagues [51] have also found that the correlation between toxic exposure and mortality was more evident in the older cohort and was stronger in women than in men. However, the determinants of these findings have yet to be clarified, especially since there are other studies that present contradictory results to what was observed in this study. Of particular note, respiratory infection studies are steadily growing, especially in the past two years due to the SARS-CoV-2 virus causing COVID-19. In a recent systematic summary, it has been shown that there is multiple evidence suggesting a strong association between the increased concentration of air pollution and an interaction with respiratory viruses such as SARS-CoV-2 [52]. The researchers observed delayed recovery as well as a more advanced and lethal progression of COVID-19 on patients who were chronically exposed to air pollution. Although the study was conducted during the phase when the pandemic was still relatively new and available information on the virus was scarce, such observations suggest that the interaction between pathogens and pollutants should be an important focus for future research given the likely negative synergistic effects.

Neurological disorders in children undergoing DNT result from clear molecular alterations found in their neuropathology. To begin with, as mentioned earlier, one of the entry points into the body for PM is through the nasal cavity. Having the advantage due to their size, pollutants such as UFPM can accumulate in the epithelium of the nasal structures of children which can translocate first into the olfactory bulb and then into the frontal cortex of the brain where UFPM can induce damage in the neurovascular unit (NVU) [39,53]. A body of data indicates Alzheimer’s (AD) and Parkinson’s (PD) as the prevailing conditions underlying the destructions of NVU. Plaque accumulation of beta-amyloid (Aβ) and hyperphosphorylation of tau (HPτ) are some of the known hallmarks of AD and PD that surprisingly have been observed during the first two decades of life of children in MCMA [54,55]. In particular, in one of the studies, 44 children living in MCMA had undergone neuropathological tests to evaluate the aggregation of four abnormally folded proteins: tau, beta amyloid, α-synuclein, and DNA-binding protein TAR (TDP-43) [55]. Microscopic images of brain sections taken from subjects demonstrated clear accumulation of amyloid plaque and hyperphosphorylated tangles. Indeed, 23% of children showed signs compatible with the development of AD and PD while 18.7% showed signs compatible with TDP-43 pathology. Similarly, a recent study by Calderón-Garcidueñas and colleagues showed that carriers of the apolipoprotein E (APOE) alleles are at high risk for AD [55]. This discovery fosters a deeper understanding of the already growing complexity of neurotoxicants, especially in the population of megacities. At the same time, recognizing APOE as a genetic factor added to other risk factors for AD can be of enormous help in advancing our knowledge on how to better protect compromised individuals from aggravating the disease. APOE as a risk factor combined with high PM exposure and a vulnerable developing nervous system should place the younger demographic sectors as top priority intervention targets for public health measures.

In addition to AD, in adults and the elderly, exposure to traffic-related particles is also linked with cases of major neurodegenerative diseases such as multiple sclerosis (MS), dementia, and Parkinson’s disease in Ontario, Canada. In a study conducted by Chen and colleagues [56], subjects who lived near Ontario’s main roads were divided into two population-based cohorts depending on the onset of the disease. Residents aged 20–50 were the multiple sclerosis cohort as MS onset predominates in individuals under the age of 50, while the PD/dementia cohort was aged 55–85, since Parkinson’s disease and the onset of dementia are mainly seen in people older than 55. The results showed that among the 4.4 million subjects in the MS cohort, 9247 cases of MS were accounted for. On the other hand, 2.2 million subjects comprised the PD/dementia cohort in which 31,577 cases were classified under the PD diagnostic category and 243,611 reported episodes of dementia. The results of statistical modeling showed that residents living in close proximity to heavy traffic were more likely to experience an increase in dementia episodes. However, no evidence was observed that associated the incidence of PD and MS with proximity to traffic. The findings from the Ontario study are relatively similar to those from Seattle, Washington, conducted by Shaffer and colleagues [57]. In their article, urban and suburban seniors who were ≥65 and had not previously been diagnosed with dementia were evaluated based on their residential areas. The result of the study shows that among the 4166 participants, 1136 were reported as incidental dementia cases. More importantly, based on four-decade data, the researchers also determined that a 1 μg/m^3^ increase in mean PM_2.5_ concentration over the period of a decade was linked to an increased risk of dementia for all causes by 16%. The extensive data on exposure to the Washington study undoubtedly shows that PM can profoundly influence the development of neurodegenerative diseases.

Recurring updates on articles and publications involving neurodegeneration help us understand the complex mechanisms behind each disease. For example, to this day, researchers still find the underlying etiology of Parkinson’s intricate. Due to epidemiological investigations, our understanding of PD is predominantly centered on its genetic and environmental factors. A large amount of data points to the idea that the pathophysiology of PD involves the accumulation and abnormal folding of the α-synuclein protein. Various mechanisms have also been proposed by which atmospheric particulate matter increases the risk of PD. To name a couple, these tiny particles can reach the brain via diffusion into the bloodstream and olfactory system [58]. Returning to the related influences of toxic particles in the gastrointestinal tract, it is puzzling how this mechanism can also promote synuclein pathology. As stated earlier, a large body of evidence proposes an association between ingested neurotoxicants and intestinal inflammation. Interestingly, this inflammation can result in a leak in an individual’s intestinal system which promotes the accumulation of the α-synuclein protein [58]. Since the structures in our gut are anatomically connected to the brain stem via the vagus nerve, α-synuclein can travel directly to regions of the brain. While talking about gut health, it is also important to keep in mind the findings linking PD and gut microbiota. Earlier in this review, it was stated that our gut microbiota may be vulnerable to heavy metals as they can alter its metabolic functions. In association with PD, a recent review revealed that an alteration of the gut microbiome due to the presence of air pollution particles can cause intestinal leaks [58]. As a result, bacterial metabolites as well as virulence factors can travel freely through the bloodstream through the intestinal lining. From here, the microbiome molecules have direct access to brain regions as they are able to traverse the BBB and induce neuroinflammation. Currently, this is one of the proposed mechanisms that may explain why increasing evidence of altered microbiome is being demonstrated in patients with PD [58].

Aside from possible neurological disorders, the cognitive abilities of children exposed to high PM concentrations are also a major focus of study in neurotoxicity research. At this point, it is relatively evident that neurotoxicants indeed have a huge impact on the developing brain. The extent of PM influence can be best interpreted by comparing subgroups living in two distinctly polluted communities. The already mentioned classic study of clinically healthy children residing in a highly polluted MCMA environment showed an increase in deficits in working memory, executive function, and fluid cognition compared with children living in a less polluted community [49]. This type of finding is of concern as a deficiency in these functions is linked to a lower IQ. For this reason, children may tend to have lower educational achievement, in the long run, their jeopardized school performance can also affect many other aspects of their work, careers, and future livelihoods as adults.

**Figure 3:**
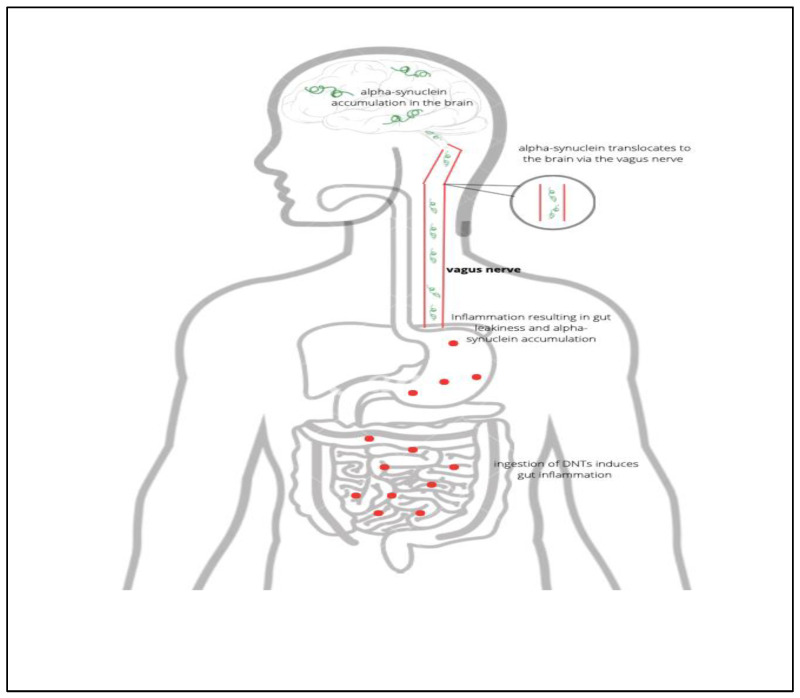
Ingestion of air pollutants leading to alpha-synuclein accumulation in the brain. Graphical illustration idea was adapted from [58], with permission from Wiley Online Library, 2022.

All in all, it seems reasonable to preliminarily conclude that neurotoxicants play a rather direct role in inducing neuroinflammation which results in various cascade systemic disorders such as complications in the enteric, respiratory, and nervous systems. Although the evidence discussed in this synthesis includes only a fraction of the massive neurotoxicity research, not only do they update our perspective and knowledge on the complexity underlying the action and impact of neurotoxicants, but they also allow us to confirm that some sectors of pediatric populations, with particular demographic and genetic characteristics, are highly vulnerable and should be given priority for intervention measures. This conclusion is supported by the following rationale: Although the developmental nature of the AD, PD, and MS and the completeness of the determining processes and mechanisms underlying such diseases are still *not* definitively proven, there is, nevertheless, sufficient evidence to warrant a *precautionary approach* [5] to protect brain development in children. Consequently, in the next section, we zoom in on a particularly vulnerable population: young children (Figure 3).

## 6. Outcomes Related to Neurotoxicants in Young Children

As mentioned above, children’s cognitive functions are strongly influenced by neurotoxicants. MCMA is one of several megacities whose youngest demographic captures the most serious consequences of neurotoxicants. However, corresponding observations of impaired cognitive abilities are also observed in individuals residing in smaller cities (<1 M population). Recent population statistics estimate 696,900 people in Boston, Massachusetts [2]. Compared with megacities such as the MCMA, Boston’s rough figures are apparently scaled down. Despite this, studies show that there are clear reports of cognitive impairments associated with air pollution in the U.S. city area. Black carbon is a known component of the PM of air which also makes it a good indicator of atmospheric pollution emitted by combustion [59]. The memory, verbal, and non-verbal skills of Boston children have been found to correlate with exposure to black carbon [60]. Parallel to other neurodegenerative disorders, cognitive impairment is not subject exclusively to a single factor. Several variables such as genetics add to its etiology. An example of this is demonstrated in relation to the aforementioned APOE allele carriers who are at higher risk for AD [61]. In addition, in many of these cases, people’s SES establishes a significant interacting impact on the degree and occurrence of these conditions. This notion is exemplified by a study conducted on black carbon exposure in the Puerto Rican cohort in the Greater Boston metropolitan area. The results suggest that impaired cognitive abilities may be observed in Puerto Ricans after long-term exposure to black carbon, compared with non-Hispanic whites [62]. The reasons for the cases of cognitive impairment in a specific cohort are not fully elucidated nor isolable. However, Wurth and colleagues propose that risk factors such as cardiovascular disease and diabetes may act as contributors since, in particular, a high prevalence of these diseases is observed in Puerto Ricans [62]. Observations on the aggravation of air pollution in children of racial/ethnic minorities are also increasing. It has previously been inferred that African American, Hispanic, or Asian children are more likely to attend high-risk public schools due to proximity to industrial sites [62]. Specifically, Grineski and Collins [63] showed that SES plays a direct role in the differences in repercussions of DNT between distinct cohorts. However, it is crucial to note that neurotoxicants are not just a concern for lower-end groups of the socioeconomic spectrum but rather that disadvantaged demographic groups bear the worst deleterious influences of neurotoxicants.

The strictly more behavioral aspect of the literature was mainly focused on the autism spectrum (ASD) and its association with exposure to neurotoxicants. In the United States, elevated levels of PM_2.5_ and UFPM, particularly concentrated in residential areas near the highway, are linked to an increase in ASD diagnosis data [34]. A shared mechanism of inflammation is thought to exist between ASD and PM of the air. The experimental results of the exposure of rodents to PM also converge with the human results. Recently published articles also support the same notion that exposure to air pollutants is linked to the incidence of ASD. A survey conducted at Kaiser Permanente hospitals in Southern California between 2001 and 2014 showed that exposure to air pollution near the highway road exposes the baby to an increased risk of ASD already in the in utero stage [64]. In addition, significant data were observed from in utero exposure to non-highway sources including road emissions and local arteries. Unfortunately, current data go even further than just indicating the association between ASD and outdoor pollution. In Shenzhen, China, autistic-like behaviors were found in children whose mothers were exposed to indoor air pollution during pregnancy [65]. Most sources of pollution come from cooking oil fumes, home decor, incineration, etc. Furthermore, the researchers also established that the degree of exposure to these indoor pollutants heavily impacts the risk of preschool-age children’s behaviors. Data suggest that exposure to multiple sources of pollutants during pregnancy puts the baby at a high risk of exhibiting autistic-like behaviors.

## 7. Magnetic Resonance Imaging (MRI) and Developmental Neurotoxicity

Behavioral and developmental deficits are now identifiable thanks to a rich repertoire of clinical evaluations. Surprisingly, the progress of science has allowed the creation of apparatuses such as magnetic resonance imaging (MRI), which connects the structure and function of the organs of our body, in this case, pertinent to the CNS. The MRI scan allowed the researchers to first discover that Mexico City children exhibit prefrontal white matter hyperintensity (WMH) in association with worsening air pollution conditions in the MCMA [66]. WMHs, also known as leukoaraoisis, are changes in areas of the brain that appear brighter, compared with other regions, as seen on an MRI. These brightly lit spots are brain lesions that are found to be more prevalent in older individuals as well as in patients with histories of dementia, cerebrovascular disease, and neurodegenerative-related cognitive impairment. Additionally, WMHs are also considered to be determinants of executive function and degeneration of cognitive performance, dementia, stroke, and even death [67]. Many clinical observations found that individuals with WMH are often asymptomatic, and in a study of clinically healthy children residing in a highly polluted environment, cognitive deficits were associated with compromised temporal and parietal lobes and global brain volume [68]. In addition to the reported hyperintensities, white matter volume was also found to decrease in children and the elderly after long-term exposure to traffic-related air pollution (TRAP), whereby TRAPs used in this study included mainly PM_2.5_ and polycyclic aromatic hydrocarbons (PAHs) [69]. Although data suggest that various neurotoxicants follow different pathways within the body, based on these cases, it is safe to assume that the degeneration of brain regions are evidentiary consequences of neurotoxicants.

A study conducted in Barcelona by Pujol et al. [70] established that children exposed to urban pollution exhibited impaired functional integration and stimulus-driven mental operations which both indicate slow brain maturation compared with normal control peers. Researchers used elemental carbon and nitrogen dioxide (NO_2_) as vehicle emission indices. Barcelona’s current population is much smaller than that of MCMA, however, the obvious damage to children’s brain structure and function is still significant. In addition, Barcelona’s air pollution conditions near the city’s schools showed moderate to high levels, compared with other areas. MRI scans also demonstrated that TRAP is highly linked to weak connectivity between the default mode network (DMN) comprising the medial prefrontal cortex, angular gyrus, and posterior cingulate cortex. DMN is relevant in this context as it identifies being active during cognitive and mental processes such as memory and attention. These processes participate in the development of children’s capacities, both academically and generally, once again creating a clear connection with the decline in cognitive abilities.

In the literature on cognitive impairment that are related to neurotoxicants, the effects on prenatal and fetal development are scarcely studied. An exception is an investigation in New York City which focused on the relationship between neurotoxicant exposure in the fetus and subsequent developmental outcomes. It was established that traces of PAHs were detected during the third trimester of pregnancy following prenatal exposure to high levels of air pollution [71]. This finding was longitudinally related to later developmental periods in the same individuals in the studied cohort in that children and youth were at higher risk of behavioral and cognitive impairments that were identified through exhibited ADHD symptoms and slower cognitive processes. Behaviors related to altered self-regulation and executive functions ranging from verbal and physical aggression to theft were also observed.

The aforementioned behavioral disturbances in New York children were somewhat similar to observations made in a study conducted in Rotterdam, the Netherlands. Researchers studied the resulting exposure to fine particles during fetal life in the finer cortices of the mid-frontal precuneus and rostrums [72]. Thinning of the cortices in these brain areas is associated with impulsivity and impaired inhibitory control. Consequently, later in life, children and adolescents tend to lose self-control in resisting the temptations that can be related to drugs, gambling, etc. Aside from addiction problems, impaired inhibitory control has also been linked to many other mental health and behavioral problems.

Cognitive and behavioral assessments and MRI scans have been extensively used for investigations into the consequences of neurotoxicants and in neurodegenerative diagnoses. The evidence in the literature offers a clear elucidation of the correlation of cognitive and behavioral disturbances with high trace amounts of PM in the air. Furthermore, the established link between exposure to neurotoxicants during the prenatal phase and the alterations occurring later in young people and adolescents may suggest that this should be the next focus of research on neurodevelopment and neurotoxicity.

## 8. Neuronal Apoptosis and Inflammation

The immense amount of literature dealing with air pollution focuses on PM, UFPM, PAH and other organic compounds. It is indisputable that the frequently studied neurodevelopmental toxic substances (such as neurotoxicants) are only a small fraction of the ever-increasing amount of toxins. Although informative, the practicality of the facts and observations of these studies is controversial. The reasons behind this is that DNT research is often ambiguous and investigating every single chemical is insurmountable and far-fetched. Thus, the most trivial conclusion is that there is no definite determination of the single or overall mechanism of action and cumulative consequences of neurotoxicants that can be conclusively accepted as ecologically valid. Of course, this does not mean that it is not possible to build a basis for defining the risks and impacts of the DNT following precautionary and preventative principles [5].

A recent synthesis of the literature has proposed a framework that explores endogenous and exogenous exposure that are partially dependent of genetic factors [7]. The Center for Disease Control has defined exposomes as a measure of aggregate exposures to individuals [73]. These exposomes can range from lifestyle choices and socioeconomic status to pre-existing infections and diseases. Endogenous sources include hormones, inflammation, oxidative stress, and metabolism. Combining both sources, the consequences of neurotoxicants can be delineated as disruptions of brain development resulting from frequent and/or long-term involuntary exposure to pollutants [5]. As a result, younger cohorts are more likely to be prone to permanent neurodegenerative disease during the later developmental period as adults.

Neuronal apoptosis is a cellular mechanism characterized by programmed cell death and is necessary for the development of the nervous system [74]. For example, the formation of synaptic connections in a developing brain resulted in the death of post-mitotic neurons. In general, apoptosis is of fundamental importance for maintaining homeostasis in the tissues. Like any other cellular mechanism, there are advantages and disadvantages attributable to cell death. For one, induction of apoptosis allows the elimination of cells that could potentially become dangerous, while excluding and preserving healthy cells. However, apoptosis could also occur uncontrollably, in which case the tissues are damaged. In a separate scenario, low levels of apoptosis could result from a buildup of damaged cells [75]. Apoptosis is often times mistaken for necrosis. It is important to note, however, that necrosis is associated with inflammation but is not triggered by apoptosis. However, toxic substances capable of promoting apoptosis can potentially induce the programmed process during critical periods of neurological development. This can be harmful as it could alter mechanistic pathways and cause behavioral and cognitive impairment.

Zhu and colleagues [76] recently determined that although there is insufficient evidence linking exposure to PM_2.5_ to neuronal damage due to cell death, neuronal apoptosis is still believed to be involved in the development of neurodegenerative diseases. S-adenosylmethionine decarboxylase 1 (AMD1) is a rate-limiting enzyme that plays a role in regulating cell apoptosis by decreasing the occurrence of the process itself. Furthermore, spermidine, a polyamine compound that has a close relationship with AMD1 through metabolism, has previously been shown to decrease neurodegeneration. In the investigation, PM_2.5_ exposure was found to cause neuronal apoptosis after damage to the cells’ mitochondria. While these two compounds are not necessarily central to our synthesis, it is a further advance that recent results reveal the relationship between AMD1 and spermidine at PM_2.5_ as these findings could potentially lead to ways to save reduced cell viability and inhibit neuronal apoptosis induced by exposure to PM_2.5_ [76].

The mechanism behind neuroapoptosis is still somewhat unclear in studies discussing its role in neurotoxicity. However, the idea that programmed cell death increases the risk of various neurodegenerative diseases [77] is a useful starting point for directing research towards a more precise definition of processes within the body. Numerous studies propose that neuroinflammation may be related to the induction of apoptosis. For example, in a recent study conducted in Mexico City, it was found that the length of telomeres in the umbilical cord blood of a fetus was reduced in association with exposure to air PM by the mother during early pregnancy [78]. Telomere length identification was successfully performed by extracting leukocyte DNA from cord blood which was then further analyzed using polymerase chain reaction. Interestingly, the researchers also found that when pregnant women were exposed to PM_2.5_ during late pregnancy, the telomere length was longer. All in all, neuroinflammation may play a role in apoptosis as we know that altered telomere length is an indicator of biological aging [7]. More specifically, oxidative stress which is part of systemic neuroinflammation induces damage to the DNA which then determines telomere length. These studies suggest that neuroinflammation should be the next goal of neurotoxic research in further investigations into the relationships between air pollution and neuronal apoptosis.

## 9. Evidence That Improvement Is Possible and Has a Critical Impact on Health and Neurological Outcomes

### 9.1. Drastic Rapid Air Pollution Reduction during COVID-19

Implementing solutions to the growing problem of environmental health on a global scale is no easy task, however, the COVID-19 pandemic provided excellent evidence of the enormous impact of global-scale interventions on environmental health achievable in a very short period. Singh and colleagues [79] showed the effect of a total reset in India on air quality. It was found that there was a 34.52% and 27.57% decrease in PM_2.5_ concentration in Calcutta and Delhi, respectively. Similarly, in a study by Stratoulias and colleagues [80], air quality data were collected following a lockdown in the city of Hat Yai, Thailand. It was found that there was a substantial drop of 33.7%, 21.8%, and 22.9% in the concentrations of NO_2_, PM_2.5_, and PM_10_, respectively. These examples (and about a dozen similar cases have emerged in the literature over the past two years) of large-scale improvement are difficult to replicate as many different factors play a role in implementing solutions nationwide. Clearly, it will be unlikely that air pollution reduction across the world will be achieved again through planned national lockdowns. However, the experience of COVID-19 has consolidated the notion that rapidly implemented changes made at the individual or community level, such as social distancing, do have a tremendous combined impact at the large-scale level. This may not suggest what would be the best and most feasible or sustainable alternatives to lockdown in the future; however, it shows *how* the alternatives will be most likely implemented. For example, switching from motorized transportation, such as cars, buses, and motorcycles, to active transportation, such as walking or cycling, can reduce air pollution and carbon dioxide emitted from vehicle exhaust. Individually, the impact is small, but the more people start implementing these small changes in their daily lives, the greater the impact on the environment and health. It is imperative that we start taking action and addressing these local and global issues immediately as the continuing global population increases and urbanization threaten the environmental health of the global population.

### 9.2. Hormetic Dose-Response, Advances towards Possible Pharmacological and Dietary Interventions

Veering away from the concept of individual and community level changes, there also exists evidence which highlights various compounds that are found to reduce occurrences and progression of some neurodegenerative diseases. As previously stated, two known hallmarks of AD include accumulation of amyloid β-peptide and hyperphosphorylated tau protein. Moreover, another indication of AD occurrence is a dysfunction in the process of reduction and oxidation of reactions within cells, a process most commonly known as redox homeostasis. It has also been observed that in patients with AD, the expression of the transcription factor nuclear factor-erythroid 2 derived factor 2 (Nrf2) was reduced and that Nrf2-related pathways, i.e., antioxidant response elements (AREs), were altered [81]. Due to these findings, researchers were able to conclude that Nrf2 and the hormetic dose-response plays a critical role in redox homeostasis and may even serve as a foundational concept towards treatment of AD. A recently published article discusses sulforaphane (SFN), a compound that is commonly found in broccoli, and its clinical relevance [82]. Researchers have revealed that the compound SFN enhances the expression of the transcription factor Nfr2 which could decelerate the onset and progression of AD [83]. In addition, utilization of an AD cellular model, Swedish amyloid precursor protein (N2a/APPswe cells), has been observed to house reduced DNA methylation levels of the Nrf2 promoter, hence upregulating the expression of Nrf2 following subjection to SFN [83]. This finding is particularly relevant as exposure to neurotoxicants has been linked to several cases of AD; thus, SFN’s capacity to serve as an anti-inflammatory and antioxidant could provide a stepping stone towards future research directions in toxicological studies.

The preconditioning signal leading to cellular protection through hormesis (mitohormesis) appears to be an important redox-dependent aging-associated feedback reaction to free radical species accumulation and inflammatory responses involved in the pathophysiology of a number of inflammation-driven disease states [84,85]. As reviewed, the generality of the hormetic dose response may extend to include hormetic nutrients as powerful chemical activators of Nrf2 and, therefore, have potentially important public health and clinical implications for DNT as well. Critically, a class of mechanisms similar to the Nrf2/ARE pathway may explain why not all children or individuals exposed to severe air pollution develop neuropathology. A large number of agents that display hormetic dose responses and at the same time activate Nrf2 can function to limit age-related damage, the progression of numerous disease processes, and chemical- and radiation-induced toxicities. Although the quantitative features and underlying mechanisms are still being investigated and are not completely known, it can be speculated that particular histories of exposure and diet associated with individual exposomes may mediate the activation of neuroprotective pathways. Current biomedical approaches have begun to elucidate a network of pro-survival pathways such as Nrf2/ARE which, under the control of protective genes (vitagenes) [86,87,88], produce molecules (heat shock proteins, glutathione, and bilirubin) endowed with antioxidant and antiapoptotic activities. The possible biological implication of these networks is in providing protective and resilience mechanisms underlying pathophysiology of oxidative, stress-driven, dysmetabolic as well as neurodegenerative diseases. Hence, disentangling the interplay and coordination of redox interactions with endogenous and exogenous antioxidant defence systems is an emerging area of research interest in anti-inflammatory antidegenerative therapeutics. This area of research may have enormous promise in combatting the deleterious effects of air pollution through the additional tools of pharmacological and dietary interventions.

## 10. Conclusions

In this research synthesis, we established the role of neurotoxicants in inducing neuroinflammation which evidently results in behavioral deficits, neurodevelopmental disorders, and neurodegeneration in cohorts of younger individuals. In general, the complexity of neurotoxicants creates an unimaginable pool of potential pathways, mechanisms, and consequences that follow after exposure. Currently, our best knowledge depends on graphical abstracts, recent publications, test data and IQ questionnaires, clinical evaluations, neurophysiological data, and MRI results. Regardless, one thing can be concluded for certain based on the literary evidence used in this analysis and the vast collections of research synthesis: children in highly urbanized and industrialized areas are the most vulnerable to pollutants and should, therefore, be given priority, as shown through the evidence of public health risks in megacities.

As established in systemic inflammation after neurotoxicant exposure, pollutants target a large variety of functional systems within the body. The repercussions on gut health and lung systems in humans and animal models demonstrate the extent of the criticality caused by these hazardous substances. Furthermore, findings suggesting a direct connection between the compromised gut and respiratory inflammation to various brain regions suggest that this should be studied in more depth as neurodegenerative diseases can result from these vulnerabilities. More critically, the similarities in the degeneration pattern in the younger and older cohort establish a critical assumption that the link between neurotoxicant exposure and neurodegeneration show developmental continuity because the effects of air pollution that are already detected early in childhood become even more evident with aging. For a paradigmatic example, the link of PM exposure to the incidence of reduced hippocampal volume was documented in four studies involving adults and two studies involving children [89]. The data from this meta-analysis show that in adults there is a strong and inverse association between PM_2.5_ concentration and hippocampal volume. Furthermore, although the correlation is weak, the results of one of the studies establish a link between PM_10_ and also the reduced volume of the hippocampus. Although Balboni and colleagues found no significant association between hippocampal volume and PM exposure in children, a recent additional source provided the missing corroborating support. Pregnant women and children in Rotterdam, the Netherlands, underwent an MRI assessment in which women were exposed to median air pollution exposure concentrations of 34.1 μg/m^3^ NO_2_ and 16.8 μg/m^3^ PM_2.5_ while the children were subjected to 32.4 μg/m^3^ NO_2_ and 16.7 μg/m^3^ PM_2.5_ [90]. These MRI results showed altered size in some very important brain regions such as the corpus callosum and the amygdala. More importantly, the investigation also found that exposure to these toxic particles during pregnancy and early childhood was associated with a volume reduction in the hippocampus as indicated by Balboni and colleagues’ study. The parallelism of size and volume of the hippocampus between the studies conducted by Balboni’s and Lubczyńska’s research teams have enormous resonance in the context of the present synthesis given the crucial role played by the hippocampus in memory, cognition, behavior, etc. Furthermore, impairments detected in younger subjects suggest a prelude to the degeneration of memory and cognition resulting from the problems of dementia and other diseases associated with neurological aging. Regardless of the fact that MRI data demonstrating brain changes are often seen in older adults, in part attributable to their age and other comorbidities, it is still somewhat puzzling to realize that the developmental risks, as well as progression of these neurodegenerative diseases, is greater when they are also subjected to pollutants. More critically, being able to observe these changes at a young age and being able to realize that exposure to these toxic substances already in the prenatal stage greatly affects brain structures and functions should be of enormous concern for public health.

The trend of research on the issues of neurotoxicity, as well as the development of available data on the neuropathology of children, points to the idea that involuntary exposure to neurotoxicants could alter their course of development and life by bearing the consequences of these substances. Although awareness on the issues of environmental risks related to pollution are today enormous components of movements of social change, and despite the fact that an immense amount of scientific studies on neurotoxicity are constantly made increasingly more openly available to the public, in the end, continuous contributions to intervention on the environmental health of the air we breathe provided by professionals in the field has the largest practical impact on the issue. As the world evolves into a more modernized, yet more highly industrialized and highly urbanized environment, the effects of air pollution on children will be a recurring problem, as evidenced by the accumulated literature. Regardless of the disparities between the various cohorts due to different influences of endogenous and exogenous factors, we must always remember that the ultimate goal should be focused on protecting children from the aggravated consequences of neurotoxicants. Science has made and will continually establish a huge contribution towards this goal; however, larger scale intervention should be introduced, such as rapid dramatic reduction in emissions, school monitoring, and community initiatives together with possible pharmacological and dietary intervention trials. All these measures would translate the impressive body of scientific knowledge into immediate precautionary, preventive action, in opposition to the delayed and reactive passive “waiting” strategy which is currently the most prevalent attitude. We believe that the present scoping synthesis contributes a step further in making this shift more compelling.

## Figures and Tables

**Table 1:** A selected sample of existing scientific evidence on the effects of heavy metal exposure to the human brain.

Type of Heavy Metal	Finding	Exposure Outcome	Ref.
**Arsenic (As)**	Subjects from areas of high As exposure display decreased serum brain-derived neurotrophic factor (sBDNF) and lower mini-mental state examination score relative to subjects that live in areas of low As exposure.	Chronic exposure to As reduces adult cognitive function. Moreover, decreased levels of sBDNF are also indicative of possible cognitive impairment.	[20]
	Exposure to As induces several pathogenic events such as apoptosis, defective mitochondrial function, oxidative stress, inflammation, stress in the endoplasmic reticulum, etc.	Alterations made following an exposure to As concur with various factors such as clinical, biological, and pathological developments of Alzheimer’s disease.	[21]
**Cadmium (Cd)**	Exposure to Cd is highly associated to an increased production of reactive oxygen species, depleted intracellular glutathione, and an increased permeability of the blood–brain barrier.	Chronic exposure to Cd causes obliteration of neuronal synaptic branches as a result of pro-inflammatory cascade triggered by Cd-affected glial cells.	[22]
**Lead (Pb)**	Residents of Myanmar that live in close proximity to lead mining areas have been highly associated with lower literacy levels.	Evidence from the study highly associates Pb exposure to impacted neurological functions. Moreover, other evidence also suggests that high Pb exposure is linked to reduced IQ, anxiety, and aggression.	[23]
	Exposure to Pb has been associated to oxidative stress, inflammation, and neuronal death.	Poor neural development in children has been linked to lead exposure. In addition, lower blood Pb levels has also been shown to affect children’s neurobehavioral functioning.	[24]
**Mercury (Hg)**	Cross referencing the effects of mercury with factors associated to the etiology of Alzheimer’s diseases has revealed that there may be an association between the two. For instance, in mercury toxicity, neurotransmitters such as glutamate, epinephrine, acetylcholine, etc. are inhibited. This similar pattern of neurotransmitter inhibition can aso be observed in patients with Alzheimer’s disease.	The main finding in this review suggests a strong correlation between exposure to Hg and incidence of Alzheimer’s disease.	[25]
	Execution of autometallography in patients with Parkinson’s disease (PD) and without PD has revealed that mercury is co-localized with Lewy bodies. In addition, Hg is also found in neurons that are known to be affected by PD such as the oligodendrocytes in white and grey matters of the brain.	Evidence in the article is indicative of the fact that Hg exposure may play a role in Parkinson’s disease.	[26]

## Data Availability

Not applicable.
